# Evaluation of preparedness of healthcare student volunteers against Middle East respiratory syndrome coronavirus (MERS-CoV) in Makkah, Saudi Arabia: a cross-sectional study

**DOI:** 10.1007/s10389-018-0917-5

**Published:** 2018-04-14

**Authors:** Mahmoud E. Elrggal, Nedaa A. Karami, Bushra Rafea, Lama Alahmadi, Anwar Al Shehri, Ruba Alamoudi, Hassan Koshak, Saad Alkahtani, Ejaz Cheema

**Affiliations:** 1 Clinical Pharmacy Department, College of Pharmacy, Umm-ul-Qura University, Taif Road, P.O. Box 13578, Makkah, Saudi Arabia; 2 College of Dentistry, Alfarabi College, Jeddah, Saudi Arabia; 3 Clinical Pharmacy Department, Faculty of Pharmacy, Najran University, Najran, Saudi Arabia; 4 Warwick Medical School, University of Warwick, Gibbet Hill Road, Coventry, UK

**Keywords:** MERS-CoV, Knowledge, Attitude, Questionnaire

## Abstract

**Aim:**

To assess the knowledge and attitude of senior medical, dental, nursing and pharmacy students toward Middle East respiratory syndrome-corona virus (MERS-CoV) in Saudi Arabia.

**Subjects and methods:**

A cross-sectional survey using a 21-item questionnaire was conducted for a 3-month period from November 2015–January 2016 in Makkah, Saudi Arabia. The questionnaire was designed to evaluate students’ understanding and perception of MERS-CoV. An ANOVA test was used to determine the association of study discipline and academic year with the student knowledge score on MERS.

**Results:**

A total of 364 students were assessed during the study. The majority (62%) of the participants were in the 20–22-year age group. More than half (53%) were pharmacy students followed by (22%) medical students. More than two thirds (71%) of the participants were aware that MERS is caused by the coronavirus. More than half (59%) of the participants believed that MERS can be transmitted through direct or indirect contact with infected camels. A statistically significant association was reported between the study discipline and mean knowledge score (*p* < 0.0001) with medical students achieving an overall better knowledge score compared with students from other study disciplines.

**Conclusion:**

Overall, students had good knowledge about MERS epidemiology, transmission and the recommended protective measures. However, students expressed their reluctance to work in healthcare facilities with inadequate MERS infection control isolation policies.

## Introduction

Since its first detection in Saudi Arabia in 2012, Middle East respiratory syndrome-corona virus (MERS-CoV) has become a major health problem (Bermingham et al. 2012; Zaki et al. 2012). Confirmed cases of MERS-CoV have been reported across the Arabian Peninsula including Jordan, Kuwait, Lebanon, Oman, Qatar, Saudi Arabia, United Arab Emirates and Yemen as well as in Asia, Europe, Africa and North America (USA) (Zumla et al. 2015). However, Saudi Arabia has reported the highest number of cases affecting 891 people with 372 reported deaths (41.8% mortality) (Zumla et al. 2015). These findings have put Saudi Arabia at the epicenter of deadly outbreaks of MERS-CoV.

The transmission of MERS-CoV through person-to-person contact has been confirmed as one of the multiple routes of transmission of MERS outbreaks in Saudi Arabia (Assiri et al. 2013; Memish et al. 2013a, b). For example, the hospital-based emergence of MERS during spring 2013 in Al-Ahsa (eastern province of Saudi Arabia) was the result of human-to-human transmission with the spread largely suspected to occur through large droplets and contact (Assiri et al. 2013). Similarly, the majority of cases detected during the 2014 outbreak of MERS in Jeddah, Saudi Arabia, were also suspected to be acquired through human-to-human transmission in healthcare facilities (Memish et al. 2013a, b; Azhar et al. 2014). Transmission of MERS from camels to humans was the other likely source implicated in the MERS-CoV outbreak in Saudi Arabia (Azhar et al. 2014).

Mass gatherings including religious festivals and congregations can carry a potentially huge health risk not only to the attendees but also to the local population. A large congregation of people at one particular place and in close proximity provides an ideal opportunity for the importation and exportation of infectious diseases and facilitates the spread of such diseases through human-to-human transmission to not only the attendees but also the local population (Abubakar et al. 2012; Al-Tawfiq et al. 2013; World Health Organization 2008). Hajj is one such gathering that every year in Makkah, Saudi Arabia, brings together millions of Muslims with multiple ethnicities, races and cultures from all over the world (Ahmed et al. 2009). It is estimated that more than 2 million Muslims from over 184 countries perform the Hajj pilgrimage every year (Alborzi et al. 2008; Gatrad and Sheikh 2005). Furthermore, pilgrims performing Hajj sacrifice four-footed animals including camels to complete one of the Hajj rituals. Pilgrims are therefore likely to be exposed to camels, which have been reported to be carriers of MERS-CoV (Azhar et al. 2014). The convergence of millions of pilgrims at one particular place in close proximity coupled with their exposure to animal carriers of MERS-CoV puts pilgrims at a major risk of contracting MERS, as particularly shown during the outbreaks of MERS in Saudi Arabia in 2013 and 2014. Fortunately, no cases of MERS were detected in pilgrims who performed Hajj during that period (Zumla et al. 2015). Nevertheless, the threat of MERS becoming a major public health epidemic remains.

Health science students enrolled in various faculties including medicine, pharmacy, dentistry and nursing at a large public university in Makkah often volunteer their services during the Hajj season to help pilgrims. These students can help to promote public awareness and understanding of MERS and the extent of its potential threat in Saudi Arabia. However, little is known about the knowledge and perception of Saudi health science students concerning MERS (Kharma et al. 2015), and more work is required to identify any knowledge gaps. This study therefore aims to assess the knowledge and attitude of senior medical, dental, nursing and pharmacy students toward MERS in Makkah, Saudi Arabia.

## Materials and methods

A cross-sectional survey was conducted for a 3-month period from November 2015–January 2016 at Umm Al Qura University, Makkah, Saudi Arabia.

### Questionnaire design

A 21-item structured questionnaire was developed using the style and format of some of the questions used in two previous studies (Kharma et al. 2015; Khan et al. 2014). The questionnaire was designed to evaluate students’ understanding and perception of MERS-CoV. Although Arabic is the national language of Saudi Arabia, the questionnaire was developed in the English language as this is the official medium of instruction at all healthcare colleges across the Kingdom. The questionnaire was piloted among a small number (*n* = 5) of undergraduate students. The presentation and validity of the questionnaire were undertaken by experienced academic and senior pharmacy students.

The study questionnaire comprised four sections containing 21 items. Section 1 had six items that explored the demographic information of respondents including age, gender, year of study, study discipline, any healthcare provider in the family and any relatives or friends who suffered from MERS. Section 2 comprised nine items and was designed to evaluate students’ in-depth knowledge about MERS including causes, sources of transmission, mortality, clinical manifestations, prevention strategies and risk groups for MERS. The knowledge was assessed at three possible levels (yes, no, I do not know). A score of 1 was given for each correct answer. No score was given for an incorrect answer. A maximum of score of 9 was obtainable in this section. Section 3 comprised one item and aimed to gather students’ sources of knowledge about MERS. Section 4 comprised five questions and aimed to evaluate students’ attitudes and beliefs about MERS. Attitude questions were designed based on a 5-point Likert scale format (1 = strongly agree, 2 = agree, 3 = neutral, 4 = disagree and 5 = strongly disagree). Positive statements were scored on a 5 to 1 scale with ‘strongly agree’ responses yielding 5 points and ‘strongly disagree’ responses 1 point. Similarly, negative statements were scored on a 1 to 5 scale with ‘strongly disagree’ responses having a maximum score of 5. ‘Neutral’ responses were scored 3. The questionnaire was developed and distributed using Google Forms.

### Questionnaire distribution and data collection

Undergraduate students studying medicine, dentistry pharmacy and dentistry were approached and recruited through social networking websites (Facebook, Twitter and Whatsapp). Students were eligible to participate if they were in year 4, 5 or 6 of their undergraduate program. The password-protected survey links were posted on various official college social media pages. An introductory paragraph outlining the aims and objectives of the study as well as instructions to complete the questionnaire was posted along with the survey.

### Statistical analysis

The data were coded, entered and analyzed using SPSS. Descriptive statistics, frequencies and percentages were used to summarize data. An ANOVA test was used to determine the association of study disciplines and academic year with the knowledge score on MERS. *P* < 0.05 was considered statistically significant.

## Results

### Demographics and characteristics of participating students

A total of 364 students were assessed during the study. The majority (62%) of the participants were in the 20–22-year age group. More than half (53%) were pharmacy students followed by medical students (22%). Most (42%) were 4th and 5th academic year students. Just over half (52%) of the participants had a healthcare provider in the family (see Table 1).

**Table 1:** Demographics and characteristics of students

Characteristics	Category	n (%)
Age	< 20	8 (2.2)
	20–22	224 (61.5)
	> 22	132 (36.3)
Gender	Male	115 (31.6)
	Female	249 (68.4)
Study discipline	Medicine	80 (22)
	Pharmacy	194 (53.3)
	Dentistry	52 (14.3)
	Nursing	38 (10.4)
Academic year	4th	151 (41.5)
	5th	154 (42.3)
	6th	59 (16.2)
Healthcare provider in the family	Yes	190 (52.2)
	No	174(47.8)
Any relatives or friends who suffered from MERS	Yes	19 (5.2)
	No	345 (94.8)

### Knowledge of students about MERS

Overall, medical students achieved significantly better knowledge scores (15.7, SD 3.7) than students from other study disciplines (*p* < 0.0001). More than two thirds (71%) of the participants were aware that MERS is caused by the coronavirus. More than half (59%) of the participants believed that MERS can be transmitted through direct or indirect contact with infected camels (see Table 2). Regarding preventive strategies for MERS, the majority (90%) of the participants believed that wearing a face mask in a crowded place could prevent the transmission of MERS. Furthermore, 82% stated that maintaining good hand hygiene can also be helpful in preventing MERS (see Table 3). More than half (60%) of the participants reported that they heard about MERS through social media, while (54%) cited TV or radio and (41%) cited posters and brochures as their sources of information (see Table 4).

**Table 2:** Knowledge of students about MERS, n (%)

	Yes	No	Don’t know
MERS is caused by coronavirus	260 (71.4)	11(3)	93 (25.5)
MERS can be transmitted through direct or indirect contact with infected camels	217 (59.6)	48 (13.2)	99 (27.2)
MERS can be transmitted easily from person to person	290 (79.7)	26 (7.1)	48 (13.2)
There is a vaccine available for MERS	48 (13.2)	231(63.5)	85(23.4)
MERS is often fatal	246 (67.6)	55 (15.1)	63 (17.3)
Antibiotics are useful for the treatment of MERS	47(12.9)	189 (52)	128 (35.2)

**Table 3:** MERS symptoms, prevention and high risk groups

		Yes, n (%)
Symptoms of MERS infection	Fever	343 (94.2)
	Cough	271 (75.5)
	Runny nose	178 (48.9)
	Shortness of breath	242 (66.5)
	Respiratory failure	199(54.7)
	Joint pain	95(26.1)
	Diarrhea	140 (38.5)
MERS can be prevented by	Use face mask in crowds	328 (90.1)
	Maintain good hand hygiene	299 (82.1)
	Avoiding close contact with MERS-infected people	308 (84.6)
	Avoiding crowded places	258 (70.9)
High-risk groups include	Healthcare providers	266 (73.1)
	Elderly	225 (61.8)
	Male gender	21 (5.8)
	Children	170 (46.7)
	People with immune system deficiency	300 (82.4)
	Travelers	97 (26.6)
	People with chronic diseases	159 (43.7)

**Table 4:** Source of knowledge about MERS, n (%)

How did you hear about MERS?	Lecture in college	117 (32.1)
	TV or radio	198 (54.4)
	Training in hospital	95 (26.1)
	Friends/ peers	123 (33.8)
	Posters and brochures	150 (41.2)
	Social media	218 (59.9)
	Seminar	40 (11.0)

### Attitude of students toward MERS

The majority (72%) of the participants strongly agreed that educating people about MERS is important to prevent the spread of the disease. Furthermore, just over half (53%) of the participants expressed their level of concern about MERS by strongly agreeing or agreeing that they will not do their clinical rotation in a hospital without a clear MERS infection control isolation policy (see Table 5). A statistically significant association was reported between study discipline and mean knowledge score (*p* < 0.0001).

**Table 5:** Attitude of students toward MERS, n (%)

	Strongly agree	Agree	Neutral	Disagree	Strongly disagree
I believe MERS is not currently a serious public health issue	27 (7.4)	33 (9)	108 (29.7)	80 (22)	116 (31.9)
MERS symptoms often resolve with time and do not require any special treatment	27 (7.4)	28 (8)	82 (22.5)	69 (19)	158 (43.4)
Educating people about MERS is important to prevent the spread of the disease	263 (72.3)	41 (11)	31 (8.5)	8 (2)	21 (5.8)
I will not do my clinical rotation in a hospital where MERS patients are treated	37 (10.2)	44 (12)	140 (38.5)	84 (23)	59 (16.2)
I will not do my clinical rotation in a hospital without a clear MERS infection control isolation policy	121 (33.2)	73 (20)	118 (32.4)	35 (10)	17 (4.7)

## Discussion

The findings of this study suggest that overall healthcare students have good knowledge and understanding concerning MERS. The majority of the participants in this study cited social media as their source of information for MERS. Study participants’ increased use of and access to the internet to seek information have also been reported in previous studies conducted in Saudi Arabia (Al-Mohrej et al. 2017; Hoda 2016; Baseer et al. 2016). The Saudi Ministry of Health often posts educational programs on infection control on its website (Baseer et al. 2016). Such educational programs can be a very useful source for providing information to both the public and various healthcare professionals. Similarly, seminars, lectures, conferences and research symposiums can also be effective in raising awareness about MERS and other emerging infectious diseases (Khan et al. 2014).

Most of the participants correctly responded that maintenance of adequate hand hygiene was paramount in the prevention of MERS. Lack of proper hand hygiene can potentially increase the risk of MERS-associated morbidity and mortality (Brug et al. 2004). The use of personal face masks was another prevention strategy for MERS that was largely supported by the study participants. Maintenance of good hand hygiene and the use of face masks and protective equipment are some of the crucial prevention strategies endorsed by the Centers for Disease Control and Prevention (CDC) to control MERS infection (CDC 2016). Other prevention strategies highly supported by the study participants included avoidance of crowded places and close contact with people infected with MERS. The role of overcrowding of patients in initiating a potential MERS outbreak particularly in hospitals with inadequate infection control measures was also highlighted in a previous study (Memish et al. 2013a).

More than half of the participants expressed their apprehension by stating that they would not do their clinical rotation in a hospital without a clear MERS infection control isolation policy. The concern showed by participants in this study also reflects their awareness about pathogen transmission (Butt et al. 2016). Transmission of MERS infection from infected patients to healthcare professionals has been confirmed in previous studies (Assiri et al. 2013; Memish et al. 2013a, b). The Saudi Ministry of Health’s scientific advisory council has developed MERS guidelines for the safer management of MERS-infected patients (Saudi Ministry of Health 2014). These guidelines have also clearly outlined the isolation procedures and precautions for the control of MERS infection. All healthcare facilities in Saudi Arabia including the Makkah region should therefore strictly adhere to these policies to ensure the protection of not only the public but also healthcare workers.

The medical students achieved a better MERS knowledge score than their counterparts. This difference may be explained by the fact that medical students have more clinical rotations and therefore have direct contact with the patients compared with pharmacy and dentistry students. Furthermore, medical students are often engaged in public health campaigns that provide them with opportunities to improve their knowledge and understanding about potentially epidemic infectious diseases such as MERS. There is, however, a need to provide specific courses to students from other study disciplines to improve their awareness of various emerging infection trends and their respective infection control policies and procedures.

This study has some limitations. Although it suggested a possible association between the study discipline and total knowledge score of students concerning MERS, this association could be explained by the risk of confounding. No power calculations were undertaken prior to the commencement of this study. However, it could be argued that this study was a descriptive study with no hypothesis testing. In this study, participants were recruited based on their willingness and ability to participate. Therefore, the sample size used in this study was based on available resources.

## Conclusion

Overall, students had good knowledge about MERS epidemiology, transmission and the recommended protective measures. However, students expressed their reluctance to work in healthcare facilities with inadequate MERS infection control isolation policies. The Saudi Ministry of Health should ensure the strict implementation of clear isolation procedures in all healthcare facilities across the Kingdom, including in Makkah, to better utilize the services of student volunteers during the umrah and Hajj season.
